# Cadmium, Chromium, and Lead Uptake Associated Health Risk Assessment of *Alternanthera sessilis*: A Commonly Consumed Green Leafy Vegetable

**DOI:** 10.1155/2021/9936254

**Published:** 2021-05-17

**Authors:** W. M. Dimuthu Nilmini Wijeyaratne, E. A. Charuni Sewwandi Kumari

**Affiliations:** Department of Zoology and Environmental Management, Faculty of Science, University of Kelaniya, Kelaniya, Sri Lanka

## Abstract

Green leafy vegetables are becoming increasingly popular in the developing countries due to their high nutritious value, common availability, and low cost. However, no studies have assessed the health risks associated with consumption of fresh green leafy vegetables. The present study assessed Cd, Cr, and Pb associated health risks in a commonly consumed green leafy vegetable in developing countries, *Alternanthera sessilis*. The Cd, Cr, and Pb concentrations in roots, leaves, and root zone soil of *Alternanthera sessilis* harvested from organic and non-organic cultivations were measured. The results indicated that Cd, Cr, and Pb concentrations in roots and leaves of *Alternanthera sessilis* exceeded the WHO/FAO safe limits for human consumption. Further, bioconcentration factor, soil to root, and root to leaf translocation factors indicated a potential of hyperaccumulating Cd in roots and leaves of *Alternanthera sessilis*. However, the target hazard quotients for Cd, Cr, and Pb were less than 1 indicating negligible health hazard associated with long time consumption of *Alternanthera sessilis*.

## 1. Introduction

*Alternanthera sessilis* leaves are rich in protein and therefore they are eaten raw as a fresh green leafy vegetable in many countries of South Asia [1]. Leaves and roots of this plant are widely used in ayurvedic medicine to treat eye and intestinal diseases as well. In Sri Lanka, *Alternanthera sessilis* is becoming an increasingly popular green leafy vegetable in everyday diet of middle-class families due to its high nutritious value and availability at low cost. With increasing consumer demand, *Alternanthera sessilis* is cultivated by both organic and non-organic cultivation practices.

The North Central province is the largest agricultural province in Sri Lanka and there are many organic and non-organic sites that cultivate *Alternanthera sessilis* in commercial basis. Despite being the largest agricultural province, this area is considered to be the highest risk area in the country for Chronic Kidney Disease with unknown etiology (CKDue). Since the first record of CKDue in Sri Lanka in the early 1990s, the number of patients has been rapidly increasing in the North Central Province and several patients have also been recorded from other areas of the country during the past decade [2]. CKDue is caused due to chronic exposure and cumulative effects of elevated levels of Cd, Cr, and Pb associated with agricultural activities [3]. According to the WHO report, one hypothesis for the cause of CKDue is presence of Cd, Cr, and Pb in the vegetables grown in the North Central Province in Sri Lanka. But the only “vegetable” analyzed in the WHO study was lotus root [4]. Further, the only heavy metal analyzed in relation to CKDue is Cd [4]. Moreover, significantly higher Cd concentrations have been recorded in tobacco harvested from CKDue prevalent areas compared to that of absent areas. However, health risks associated with consumption of these high Cd containing products have not been quantified in these studies [5, 6]. Further, even though the green leafy vegetables are an essential food item in everyday meals, no research has been conducted to assess the concentration of Cd, Cr, or Pb in the green leafy vegetables harvested from CKDue prevalent areas.

Therefore, the present study focused on assessment of Cd, Cr and Pb concentrations in the roots and edible portions of the most commonly consumed green leafy vegetable in Sri Lanka, *Alternanthera sessilis* harvested from organic and non-organic cultivations in CKDu prevalent areas in the North Central Province.

The aim of the study was to assess the potential health risks associated with the consumption of organically and non-organically grown *Alternanthera sessilis* in terms of bioconcentration factor (BCF), soil to root translocation factor (TF(soil − root)), and root to leaf translocation factors (TF(root − leaf)), target hazard quotient (THQ) for each heavy metal and hazard index (HI).

## 2. Materials and Methodology

Four *Alternanthera sessilis* cultivation sites (non-organic sites: (80°46′22.678″E, 8°53′48.032″N) and (80°45′58.693″E, 8°52′42.93″N); Organic sites: (80°47′12.361″E, 8°51′34.401″N) and (80°48′56.867″E, 8°53′20.621″N)) with an area of 100 m^2^ each were selected as study sites from Padaviya area in the North Central Province in Sri Lanka. The distance between any two sampling sites was about 10.5 ± 0.2 km. Each cultivation site was divided into 2 × 2 m^2^ sub-plots. At the end of each culture cycle during dry (April 2019) and rainy seasons (October 2019), twenty plants and their root zone soil samples were collected randomly from each sub-plot. The sampled plant and soil samples were transported to the laboratory. In the laboratory, the plants were washed with double distilled water and were separated into leaves and roots. The roots and leaves were oven-dried at 70°C until they attained a constant weight. The dried leaf and root samples were ground into powder using a mechanical grinder and then sieved through 0.425 mm mesh size sieve. The sieved samples were stored in desiccators until they were acid-digested. The soil samples were oven-dried at 105°C for 24 hours and then, ground into a fine powder using acid washed commercial mortar and pestle, and sieved through 0.425 mm mesh sieve to separate the bio-exchangeable fraction of the heavy metals [7]. The sieved soil samples were also stored in the polyethylene bags and placed in desiccators until they were subjected to acid digestion [7].

The powdered sieved soil samples were acid-digested using EDTA (Ethylene Diamine Tetra acetic acid) in the Kjeldatherm digestion system. The powdered sieved plant samples were acid digested using Conc HNO_3_ acid in the Kjeldatherm digestion system. The acid-digested samples were analyzed for Cd, Cr, and Pb using an atomic absorption spectrophotometer (Analytic jena Model novAA 400p) on graphite furnace mode following the procedure described in APHA [8]. The minimum detection limits were 0.02 mg/L for all the analyzed metals. Sandy loam certified reference material CRM 023 (Sigma Aldrich, USA) was used as standard reference material for soil analysis and white cabbage certified reference material BCR 485 (Sigma Aldrich, USA) was used as standard reference material for plant part analysis. Continuing control verification was done after every 10 samples to check that variability was within 10%.

To determine the accuracy of the methods used in the determination of the concentration of the metals in the soil, root, and leaf extracts, the soil, root, and leaf samples were spiked with known-amounts of the elements studied (Cd, Cr, and Pb). The recovery values (%) for each metal were calculated by comparing the concentration of spiked samples with non-spiked samples.

Electrical conductivity, pH, cation exchange capacity, and organic matter content of soil collected from each sampling plot at each sampling season were analyzed in the laboratory following the procedures described in APHA [8].

The heavy metal concentrations were used to calculate the bioconcentration factor (BCF), of Cd, Cr, and Pb as described by Sulaiman et al. [9].

(1) $$\begin{array}{c} \text{BCF}=\frac{C_{\text{Plant}}}{C_{\text{Soil}}}, \end{array}$$

where *C*_plant_ is the concentration of metal in plant part and *C*_soil_ is the concentration of metal in soil.

Soil to root and root to leaf translocation factors (TF_(soil − root)_ and TF_(root − leaf)_) were calculated using following formulas as described by Sulaiman et al. [9].

(2) $$\begin{array}{c} \text{TF}{}_{\left(\text{soil}-\text{root}\right)}=\frac{C\;{}_{\text{roots}}}{C\;{}_{\text{soil}}}, \end{array}$$

where TF_(soil − root)_ is the translocation factor from roots to leaves, *C*_leaves_ is the concentration of metal in leaves, and *C*_root_ is the concentration of metal in root [9].

(3) $$\begin{array}{c} \text{TF}{}_{\left(\text{root}-\text{leaf}\right)}=\frac{C\;{}_{\text{leaves}}}{C\;{}_{\text{roots}}}, \end{array}$$

where TF_(root − leaf) _ is the translocation factor from soil to roots *C*_roots_ is the concentration of metal in roots, and *C*_soil_ is the concentration of metal in soil [9].

The potential health risk for heavy metal consumption from *Alternanthera sessilis* was assessed by calculating the target hazard quotient (for adults and children) using the following formula as described by the United States Environmental Protection Agency [10, 11]:

(4) $$\begin{array}{c} \text{THQ}=\frac{E_{F}E_{D}F_{\text{IR}}C}{R_{\text{FD}}W_{\text{AB}}T_{A}}\times 10^{-3}, \end{array}$$

where *E*_*F*_ is the exposure frequency (156 days/year considering *Alternanthera sessilis* is included in the diet 3 days per week); *E*_*D*_ is the exposure duration (77 years, equivalent to the average lifetime of the Sri Lankan population); *F*_IR_ is the food ingestion rate (US EPA recommended average leafy vegetable consumption rates for adults and children is 2.2 g/person/day, respectively) [12]; C is the metal concentration in the edible parts of vegetables (mg/kg); *R*_FD_ is the oral reference dose (Pb, Cd, Cr, and Ni values were 0.0035, 0.001, 1.5, and 0.02 mg/kg/day, respectively [13, 14]); *W*_AB_ is the average body weight (70 kg for adults and 30 kg for children) [15]; and *T*_*A*_ is the average exposure time for non-carcinogens (*E*_*D*_*X* 365 days/year). A THQ value greater than 1 indicates that the exposure is likely to cause obvious adverse effects to human health [11].

The hazard index (HI) in the study sites was calculated as the sum of individual THQs for each metal [15].

Data were tested for normality using Anderson Darling test and the non-normalized data were log transformed. ANOVA followed by Tukey's pairwise comparison was used to analyze the variation of physical and chemical parameters of root zone soil and the heavy metal concentrations of roots, leaves and root zone soil collected from the non-organic and organic cultivation sites during rainy and dry seasons. The data were analyzed using MINITAB 17 software.

## 3. Results and Discussion

The percentage recovery of Cd, Cr, and Pb in soil samples is presented in Table 1. The percentage recovery of heavy metals in the plant, root, and soil extracts was higher than 95%.

Spatial variation of physicochemical parameters and heavy metal concentrations in root zone soil of *Alternanthera sessilis* is presented in Table 2. Conductivity, cation exchange capacity, Cr, and Cd concentrations showed significant spatial and temporal variations among study sites (Table 2). Significantly high conductivity and organic matter content were recorded during the rainy season compared to those in the dry season in both non-organic and organic study sites. Significantly high cation exchange capacity, Cr, and Cd concentrations were recorded from the soil in the non-organic sites compared to those in the organic sites during both seasons. Phosphate fertilizers and carbamate insecticides are considered as major sources of heavy metals that contaminate soil in the agricultural areas [16, 17]. Therefore, application of chemical fertilizers and chemical pest management strategies may have influenced the increased cation exchange capacity, Cd, and Cr concentrations in the non-organic sites. However, the Pb, Cd, and Cr concentrations in all the study sites were below the critical levels recommended by European Union (EU) for healthy agricultural activities (300 mg/kg for Pb; 180 mg/kg for Cr; and 6.4 mg/kg for Cd) (Table 2). Significantly high organic matter content was recorded from the soil in the organic sites compared to those in the non-organic sites during both seasons (Table 2). In the organic cultivation sites, addition of compost as a soil conditioner is frequently practiced in order to improve the soil quality and maintain a healthy moisture condition, where as in the non-organic sites composting is not practiced. Therefore, the compost application may have influenced the significantly high organic matter content in the organic cultivations.

Mean Cr, Cd, and Pb concentrations recorded from roots of *Alternanthera sessilis* sampled from non-organic and organic cultivations are presented in Table 3. The heavy metal concentration of the roots of *Alternanthera sessilis* varied as Cr > Pb > Cd. The heavy metal concentration in the roots of *Alternanthera sessilis* during dry and rainy seasons was not significantly different in both organic and non-organic cultivation sites. The concentration of heavy metals in the roots of *Alternanthera sessilis* was significantly high in the non-organic cultivation sites compared to those in the organic cultivation sites during both dry and rainy seasons (Table 3). The roots are in direct contact with the metals and nutrients present in the soil and thereby consequently uptake them from the soil solution. The heavy metal uptake by the plant roots can be affected by pH and organic matter content of soil. The high organic matter content in the organic cultivation sites may be contributing to the reduction of plant uptake of heavy metals as organic matter can adsorb heavy metals, which thereby reduce the availability of heavy metal ions to be taken up by plant roots [18]. However, the mean Pb, Cd, and Cr concentrations in roots of *Alternanthera sessilis* in all the study sites were higher than the levels recommended by European Union (EU) safe limits for consumption (0.3 mg/kg. for Pb; 2.3 mg/kg for Cr; and 0.2 mg/kg for Cd) (Table 3).

The mean Cr, Cd, and Pb concentrations recorded from leaves of *Alternanthera sessilis* sampled from non-organic and organic cultivation sites are presented in Table 4. The heavy metal concentration of the leaves of *Alternanthera sessilis* varied in a similar pattern to that of the roots as Cr > Pb > Cd. The concentration of Cd and Cr in the leaves of *Alternanthera sessilis* was significantly high in the non-organic cultivation sites compared to the organic cultivation sites during both dry and rainy seasons. However, there was no significant variation in the Pb concentration in the leaves of *Alternanthera sessilis* in both types of cultivations (Table 4).

Cr is an essential element for many biological activities in plants and is very important in protein metabolism. However, accumulation of Cr in high concentrations can result in toxic responses [19, 20]. Further, Cd and Pb are non-essential elements which cause toxic effects even at very low concentrations [16]. Nevertheless, accumulation of Cd in trace concentrations in plants affects the nutrients uptake, obstruct the respiratory enzymes, carbohydrate metabolism, photosynthesis, alter the antioxidant metabolism, and reduce the crop productivity [21]. In the present study, the studied metal concentrations in all leaves harvested from non-organic and organic cultivation sites exceeded the levels recommended by WHO/FAO safe limits for human consumption (0.3 mg/kg for Pb; 2.3 mg/kg for Cr; and 0.2 mg/kg for Cd), thereby causing potential health risks to the consumers.

**Figure 1:**
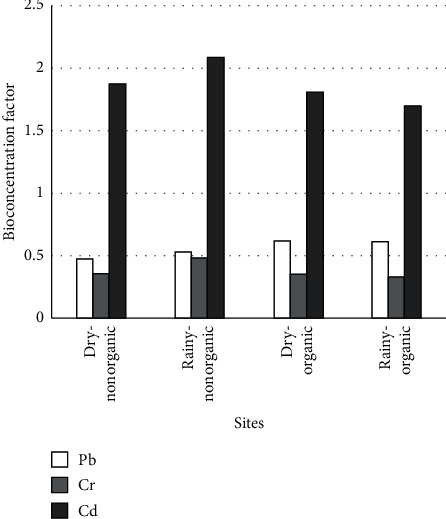
Bioconcentration factor of Cd, Cr, and Pb for *Alternanthera sessilis* in the study sites during dry and rainy seasons.

The bioconcentration factor of *Alternanthera sessilis* for Cd, Cr, and Pb in the study sites during dry and rainy seasons is given in Figure 1. The bioconcentration factor for Cd, Cr, and Pb in the non-organic sites was 1.9, 0.4, and 0.5, respectively, during the dry season and 2.0, 0.5, and 0.5, respectively, during the rainy season. Further, the bioconcentration factor for Cd, Cr, and Pb in the organic sites was 1.8, 0.4, and 0.6, respectively, during the dry season and 1.7, 0.3, and 0.6, respectively, during the rainy season (Figure 1). Bioconcentration factor (BCF) is an important indicator used in environmental toxicology and risk assessment to determine the degree of intake and storage of toxic substances in biota [22, 23]. In plants, the BCF is defined as the ratio of metal concentration in plant part to the corresponding metal concentration in soil. BCF is used to identify hyper-accumulator species for heavy metals [24, 25]. If the BCF is greater than 1 for a particular metal, the plant is considered as a hyper-accumulator of that metal [22]. According to the results of the present study, *Alternanthera sessilis* can be considered as a hyper-accumulator of Cd as the BCF for Cd is greater than 1 in all the sites during both dry and rainy seasons (Figure 1).

**Figure 2:**
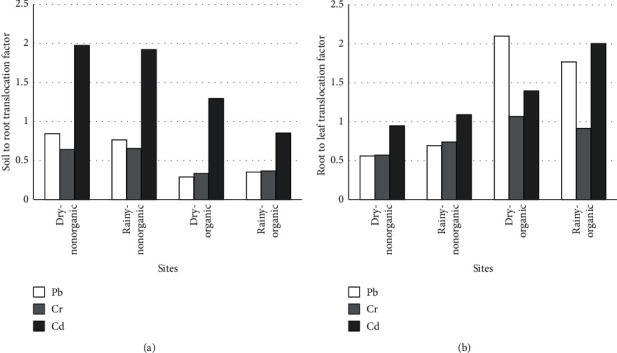
Translocation factor of *Alternanthera sessilis* for Cd, Cr, and Pb in the study sites during dry and rainy seasons. (a) Soil to root translocation factor; (b) root to leaf translocation factor.

Translocation factor (TF) can also be used as an index to assess the hyper-accumulation capacity of heavy metals in plants. TF assesses the capacity to accumulate heavy metals in the roots, stems, or leaves of the plant. [26–28]. The soil to root and root to leaf translocation factors of *Alternanthera sessilis* for Cd, Cr, and Pb in the study sites during dry and rainy seasons are given in Figure 2. The soil to root translocation factor of *Alternanthera sessilis* for Cd, Cr, and Pb in non-organic sites was 1.9, 0.6, and 0.8, respectively, during the dry season and 1.9, 0.6, and 0.8, respectively, during the rainy season. The soil to root translocation factor of *Alternanthera sessilis* for Cd, Cr, and Pb in organic sites was 1.8, 0.4, and 0.6, respectively, during the dry season and 1.7, 0.3, and 0.6, respectively, during the rainy season. According to the results of the present study, the soil to root TF for cadmium is greater than 1, indicating hyperaccumulation potential of Cd in the roots of *Alternanthera sessilis*. Further, root to leaf translocation factor of *Alternanthera sessilis* of Cd, Cr, and Pb in non-organic sites was 0.9, 0.6, and 0.6, respectively, during the dry season and 1.1, 0.7, and 0.7, respectively, during the rainy season. Similarly, in the organic sites, TF for Cd, Cr, and Pb was 1.4, 1.0, and 2.1, respectively, during the dry season and 2.0, 0.9, and 1.8, respectively, during the rainy season (Figure 2). The results of present study indicate that there is a potential of hyperaccumulating Cd in the leaves of *Alternanthera sessilis*. Further, in the organic sites, there is a potential to hyperaccumulate Pb and Cr in the leaves of *Alternanthera sessilis*. TF > 1 at root to leaves may also indicate the possibility of foliar metal absorption due to atmospheric deposition of heavy metals on GLV surfaces, in addition to uptake via the root system [29].

The target hazard quotient values (THQ) for each heavy metal and the hazard index (HI) for adults and children are given in Table 5. The sequence of THQ for adults and children ranged as Pb < Cr < Cd in both organic and non-organic sites (Table 5). The THQ for each heavy metal was less than 1 in both non-organic and organic cultivations for both adult and child populations. A THQ less than 1 indicates that there is no or negligible health hazard associated with long term consumption of *Alternanthera sessilis*. However, in the non-organic cultivation sites, HI for adult population was 1.2. A HI higher than 1 indicates that there is a potential to cause adverse non-cancer health effects over a lifetime of exposure to *Alternanthera sessilis* cultivated using non-organic practices. [30].

Heavy metal input into the agricultural fields can mainly be due to the application of chemical fertilizers and pesticides. In addition, heavy metals can be released into the groundwater when the soils reduce the heavy metal retaining capacity due to continuous loading of pollutants or changes in pH. The plants can uptake heavy metals released to the soils via root uptake mechanisms [31]. Mobilization of heavy metals in the soil is a function of pH, clay content, organic matter content, cation exchange capacity, and other soil properties making each soil unique in terms of pollution management.

Accumulation and distribution of trace elements and heavy metals in plants can be varied among by plant species and can be influenced by genetic and morphological characteristics of plants. In addition, the concentration in the medium, bioavailability of metals, their ionic state, soil characteristics (pH, organic matter, cation exchange capacity, etc.), vegetation period, climacteric conditions, and multiple other factors can also influence the accumulation and distribution of heavy metals in the plants. Continuous uptake and mobilization of metals inside plants can increase the concentration of metals in plant tissues compared to that of the soil which has relatively low metal concentrations [32]. Such results can be attributed to the root uptake mechanisms, as well as the foliar absorption potential of atmospheric metal deposits on plant surfaces [33].

## 4. Conclusion

The results of the present study indicate that *Alternanthera sessilis* has a potential to hyperaccumulate Cd in the leaves. However, according to the target hazard quotient, there are negligible health hazards associated with long time consumption of organically cultivated or non-organically cultivated *Alternanthera sessilis* by adult and child populations. Therefore, *Alternanthera sessilis* can be considered as a potentially safe green leafy vegetable for daily consumption. However, regular monitoring of heavy metals in *Alternanthera sessilis* is crucial to avoid excessive buildup of these metals along the food chain.

## Figures and Tables

**Table 1:** Percentage recovery of Cd, Cr, and Pb in the soil, root, and leaf extracts.

	Percentage recovery ± standard deviation (%)		
	Cd	Cr	Pb
Soil extract	95.6 ± 0.1	95.8 ± 0.1	96.1 ± 0.3
Root extract	96.2 ± 0.2	95.1 ± 0.1	96.1 ± 0.2
Leaf extract	95.4 ± 0.2	95.1 ± 0.2	95.7 ± 0.2

**Table 2:** Spatial variation of physicochemical parameters and heavy metal concentrations in the root zone soil sampled from non-organic and organic *Alternanthera sessilis* cultivations in the Padaviya area, Sri Lanka. Data are presented as mean ± SD. In each row, the mean values with different superscript letters are significantly different from each other (ANOVA, Tukey's test, *P* < 0.05, *N* = 80).

	Non-organic cultivation sites		Organic cultivation sites	
	Dry season	Rainy season	Dry season	Rainy season
pH	6.63 ± 0.08^a^	6.22 ± 0.36^a^	6.57 ± 0.12^a^	6.52 ± 0.09^a^
Conductivity (*μ*S/cm)	213.7 ± 22.1^a^	173.05 ± 12.4^b^	147.1 ± 19.3^c^	206.72 ± 19.4^a^
Organic matter content (%)	2.62 ± 0.41^a^	2.46 ± 1.32^a^	3.98 ± 0.33^b^	5.36 ± 1.2^c^
Cation exchange capacity (meq/100 g)	17.25 ± 1.00^a^	20.50 ± 1.00^a^	13.75 ± 0.50^b^	13.00 ± 1.00^b^
Pb (mg/kg dry weight of soil)	5.59 ± 0.62^a^	6.44 ± 0.42^a^	5.17 ± 0.52^a^	5.50 ± 0.37^a^
Cr (mg/kg dry weight of soil)	27.87 ± 3.78^a^	28.65 ± 4.28^a^	18.67 ± 2.66^b^	18.68 ± 3.52^b^
Cd (mg/kg dry weight of soil)	1.00 ± 0.09^a^	1.03 ± 0.00^a^	0.37 ± 0.01^b^	0.4 ± 0.00^b^

**Table 3:** The mean Cr, Cd, and Pb concentrations recorded from roots of *Alternanthera sessilis* sampled from non-organic and organic cultivations. Results are presented as mean ± SD. In each row, the mean values with different superscript letters are significantly different from each other (ANOVA, Tukey's test, *P* < 0.05, *N* = 80).

	Mean concentration (roots) mg/kg dry weight			
	Non-organic cultivation sites		Organic cultivation sites	
Metal	Dry season	Rainy season	Dry season	Rainy season
Pb	4.72 ± 0.55^b^	4.94 ± 1.54^b^	1.53 ± 0.11^a^	1.93 ± 0.42^a^
Cr	17.875 ± 3.29^b^	18.86 ± 1.96^b^	6.33 ± 1.71^a^	6.86 ± 1.17^a^
Cd	1.98 ± 0.72^b^	1.98 ± 0.03^b^	0.48 ± 0.01^a^	0.34 ± 0.09^a^

**Table 4:** The mean Cr, Cd, and Pb concentrations recorded from leaves of *Alternanthera sessilis* sampled from non-organic and organic cultivations. Results are presented as mean ± SD. In each row, the mean values with different superscript letters are significantly different from each other (ANOVA, Tukey's test, *P* < 0.05, *N* = 80).

	Mean concentration (leaves) mg/kg dry weight			
	Non-organic cultivation sites		Organic cultivation sites	
	Dry season	Rainy season	Dry season	Rainy season
Pb	2.65 ± 0.69^a^	3.43 ± 0.70^a^	3.20 ± 0.42^a^	3.40 ± 1.15^a^
Cr	10.10 ± 7.52^a^	13.85 ± 0.61^a^	6.70 ± 12.10^b^	6.26 ± 0.31^b^
Cd	1.87 ± 0.21^a^	2.15 ± 0.26^a^	0.67 ± 0.14^b^	0.68 ± 0.07^b^

**Table 5:** Target hazard quotient (THQ) and hazard index of Cd, Cr, and Pb in adult and child populations.

Population	Non-organic cultivations				Organic cultivations			
	THQ-Cd	THQ-Cr	THQ-Pb	HI	THQ-Cd	THQ-Cr	THQ-Pb	HI
Adults	0.7	0.4	0.1	1.2	0.3	0.2	0.1	0.6
Children	0.35	0.2	0.05	0.6	0.15	0.1	0.05	0.3

## Data Availability

The data will be made available upon request.
